# DNA damage reflected in the evolution of G-runs in genomes

**DOI:** 10.18699/vjgb-25-98

**Published:** 2025-12

**Authors:** I.R. Grin, D.O. Zharkov

**Affiliations:** Institute of Chemical Biology and Fundamental Medicine of the Siberian Branch of the Russian Academy of Sciences, Novosibirsk, Russia; Institute of Chemical Biology and Fundamental Medicine of the Siberian Branch of the Russian Academy of Sciences, Novosibirsk, Russia Novosibirsk State University, Novosibirsk, Russia

**Keywords:** DNA damage, mutagenesis, 8-oxogianine, G-runs, telomeres, повреждение ДНК, мутагенез, 8-оксогуанин, G-тракты, теломеры

## Abstract

DNA oxidation is one of the main types of damage to the genetic material of living organisms. Of the many dozens of oxidative lesions, the most abundant is 8-oxoguanine (8-oxoG), a premutagenic base that leads to G→T transversions during replication. Double-stranded DNA can conduct holes through the π system of stacked nucleobases. Such electron vacancies are ultimately localized at the 5’-terminal nucleotides of polyguanine runs (G-runs), making these positions characteristic sites of 8-oxoG formation. While such properties of G-runs have been studied in vitro at the level of chemical reactivity, the extent to which they can influence mutagenesis spectra in vivo remains unclear. Here, we have analyzed the nucleotide context of G-runs in a representative set of 62 high-quality prokaryotic genomes and in the human telomere-to-telomere genome. G-runs were, on average, shorter than polyadenine runs (A- runs), and the probability of a G-run being elongated by one nucleotide is lower than in the case of A-runs. The representation of T in the position 5’-flanking G-runs is increased, especially in organisms with aerobic metabolism, which is consistent with the model of preferential G→T substitutions at the 5’-position with 8-oxoG as a precursor. Conversely, the frequency of G and C is increased and the frequency of T is decreased in the position 5’-flanking A- runs. A biphasic pattern of G-run expansion is observed in the human genome: the probability of sequences longer than 8–9 nucleotides being elongated by one nucleotide increases significantly. An increased representation of C in the 5’-flanking position to long G-runs was found, together with an elevated frequency of 5’-G→A substitutions in telomere repeats. This may indicate the existence of mutagenic processes whose mechanism has not yet been characterized but may be associated with DNA polymerase errors during replication of the products of further oxidation of 8-oxoG.

## Abstract (ru)

Окисление ДНК представляет собой один из главных видов повреждения генетического материала живых организмов. Из многих десятков продуктов окислительного повреждения ДНК в наибольшем количестве встречается 8-оксогуанин (8-oxoG) – предмутагенное основание, приводящее при репликации к трансверсиям G→T. Двуцепочечная ДНК обладает способностью к проводимости положительных зарядов, связанных с дефицитом электронов в π-системе азотистых оснований. Такие заряды в конечном итоге локализуются на 5’-концевом нуклеотиде полигуаниновых трактов (G-трактов). В связи с этим 5’-концевые нуклеотиды G-трактов служат характерными местами образования 8-oxoG. Эти свойства G-трактов хорошо изучены in vitro на уровне реакционной способности, но остается неясным, насколько они могут отражаться в спектрах мутагенеза in vivo. В работе проанализирован нуклеотидный контекст G-трактов в репрезентативном наборе из 62 полных геномов прокариот и в геноме человека с покрытием «от теломеры до теломеры». Показано, что G-тракты в среднем короче полиадениновых трактов (A-трактов) и вероятность удлинения G-трактов на один нуклеотид ниже, чем в случае A-трактов. Установлено, что представленность T в положении, примыкающем к G-трактам с 5’-стороны, повышена, в особенности у организмов с аэробным метаболизмом, что согласуется с моделью преимущественных мутаций G→T в 5’-положении с 8-oxoG как предшественником. В то же время в положении, примыкающем к A-трактам, повышена частота встречаемости G и C и снижена частота встречаемости T. В геноме человека наблюдается двухфазный характер разрастания G-трактов: начиная с длины 8–9 нуклеотидов вероятность их удлинения на один нуклеотид заметно увеличивается. Выявлена повышенная представленность C с 5’-стороны от длинных G-трактов и A при заменах в теломерных повторах, что может свидетельствовать о существовании мутагенных процессов, механизм которых пока не охарактеризован, но может быть связан с ошибками ДНК- полимераз при репликации продуктов дальнейшего окисления 8-oxoG.

## Introduction

Oxidative DNA damage is an inevitable consequence of respiration, which relies on the oxidation of organic compounds with molecular oxygen and has been the basis of energy metabolism in the vast majority of living organisms for over two billion years (Prorok et al., 2021). Damaged nucleotides are generally quickly repaired; however, some of them may remain in DNA until replication, which is one of the main sources of mutations (Liu et al., 2016; Chatterjee, Walker, 2017; Tubbs, Nussenzweig, 2017). Based on our understanding of the molecular mechanisms of DNA polymerase errors, it has now become possible to identify characteristic patterns of mutations caused by various types of genotoxic stress or even by specific damaged bases (Alexandrov et al., 2013; Koh et al., 2021).

**Fig. 1.**
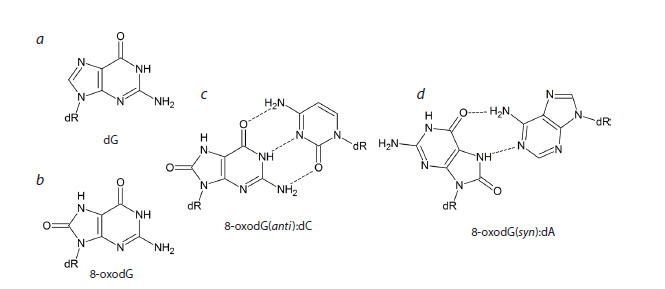
Structures of 2’-deoxyguanosine (a), 8-oxo-2’-deoxyguanosine (b), Watson–Crick 8-oxodG(anti ):dC pair (c) and Hoogsteen 8-oxodG(syn):dA pair (d).

Of all DNA structural elements, the guanine base has the lowest redox potential (Cadet et al., 2008, 2017; Fleming, Burrows, 2022). The most common product of its oxidation, 7,8-dihydro-8-oxoguanine (8-oxoG), occurs in DNA at the background level of ~1/106 guanines, and this level increases significantly under oxidative stress of various origins (ESCODD et al., 2005; Dizdaroglu et al., 2015; Chiorcea- Paquim, 2022; Fig. 1a, b). The presence of an oxygen atom at C8 in 8-oxoG sterically hinders the regular anti conformation of its nucleoside, 8-oxo-2′-deoxyguanosine (8-oxodG), and the syn conformation becomes energetically favorable (Cho et al., 1990; Fig. 1c, d ). Consequently, in the absence of Watson– Crick bonds with cytosine, which additionally stabilize the anti conformation, 8-oxodG preferentially adopts the syn conformation, in which it can form a Hoogsteen-type pair with adenine (Kouchakdjian et al., 1991; McAuley-Hecht et al., 1994; Lipscomb et al., 1995). Because of this, DNA polymerases incorporate dAMP opposite 8-oxoG in the DNA template with high frequency (Shibutani et al., 1991; Miller, Grollman, 1997; Maga et al., 2007; Yudkina et al., 2019).

In the living cell, the outcome of primary DNA oxidation events can be influenced by numerous additional factors and DNA repair systems that remove damaged bases from the genome. Even so, 8-oxoG exhibits relatively high mutagenicity in vivo, characterized by a spectrum dominated by G→T transversions mostly independent of the surrounding nucleotide context (Wood et al., 1992; Moriya, 1993). Such mutations are frequently found in human tumors and form the basis of the SBS18 and SBS36 mutational signatures (Alexandrov et al., 2013; Pilati et al., 2017; Viel et al., 2017; Kucab et al., 2019). Guanidinohydantoin and spiroiminodihydantoin, the products of further oxidation of 8-oxoG, also significantly contribute to mutagenesis, predominantly causing G→C transversions (Fleming, Burrows, 2017; Kino et al., 2020).

The stacked π system of DNA has considerable hole conductivity (Giese, 2002; Genereux, Barton, 2010). Numerous experiments and quantum mechanical calculations show that a positive charge resulting from one-electron oxidation of DNA can migrate along the π system over significant distances, and its final acceptors are the G bases, which are mainly oxidized to 8-oxoG. In this case, the G bases located in the first 5′-position in runs of several Gs are especially sensitive to oxidation (Sugiyama, Saito, 1996; Saito et al., 1998; Kurbanyan et al., 2003; Adhikary et al., 2009).

Although the mechanism of positive charge migration and preferential oxidation of guanines at the 5′-end of G-runs is generally accepted today, all experimental data supporting were obtained in relatively simple in vitro systems. The mutagenesis spectra caused by the appearance of 8-oxoG in this context have not yet been studied. If preferential conversion of G to 8-oxoG does indeed occur at the 5′-end of G-runs, it can be expected that the mutagenic properties of 8-oxoG at these positions will result in an increased frequency of G→T mutations, which should be reflected in an increased frequency of T before G-runs. In this study, to test this hypothesis, we analyzed the occurrence of nucleotides flanking G-runs from the 5′-side in prokaryotic and human genomes.

## Materials and methods

The T2T-CHM13v2.0 human genome assembly, which includes full-length telomeres and highly repetitive regions (Nurk et al., 2022), and the prokaryotic genomes listed in Table 1 were used for the analysis.

**Table 1.**
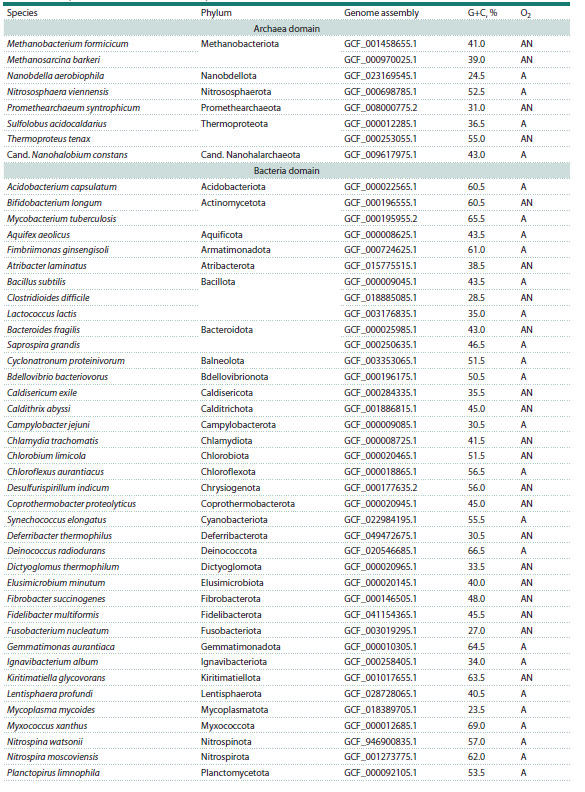
Prokaryotic genomes used for the analysis

**Table 1end.**
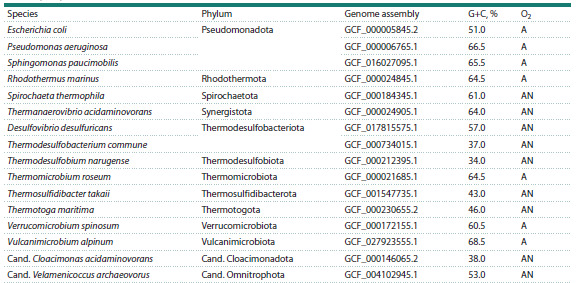
Table 1end. Note. Assembly ID in the RefSeq database (O’Leary et al., 2016). A, aerobes and facultative anaerobes; AN, anaerobes.

UGENE v37.0 software package (Okonechnikov et al., 2012) and custom-written bash scripts were used to extract nucleotide frequencies at given positions. The expected frequency of nucleotides in the flanking positions before and after Gn (or An) runs in prokaryotic genomes was calculated based on the total number of A, C, and T (or C, G, and T) in a given genome as pA = NA/(NA+NC+NT), where pA is the expected representation (in this case, for A), and NA, NC, and NT are the numbers of A, C, and T in both strands of the genome, respectively. For the human genome, due to the well-known underrepresentation of the CG dinucleotide, the expected frequency was calculated in a similar way but based on the number of AG, CG, and TG dinucleotides. Statistical analysis was performed using SigmaPlot v11.0 (Grafiti, USA), DATAPLOT (National Institute of Standards and Technology, USA), and RStudio v1.2 (Posit PBC, USA). Dunn’s correction was used for all multiple comparisons and test series to adjust the significance level.

## Results and discussion

To analyze the nucleotide distribution in prokaryotic genomes, a sample of 54 bacterial and 8 archaeal genomes was compiled, maximally reflecting the taxonomic diversity in these domains of life (Table 1). Only high-quality genomes classified in the RefSeq database (O’Leary et al., 2016) as reference genomes were included. The sample taxonomic representation was one genome per phylum, with the exception of Methanobacteriota and Thermoproteota for Archaea, and Actinomycetota, Bacteroidota, and Thermodesulfobacteriota for Bacteria with a representation of 2 genomes from different orders per phylum, as well as Bacillota and Pseudomonadota (3 genomes from different orders per phylum). The G+C content in the studied genomes ranged from 23.5 to 69 % (Table 1). The parameters of archaeal genomes did not differ significantly from those of bacterial ones, so the representatives of both domains were considered as a single group of prokaryotes.

Since the prokaryotic genomes mostly consist of proteincoding sequences, mutations in which can be subject to natural selection, we have first assessed the possible impact of all 16 potential amino acid substitutions resulting from G→A, G→C and G→T nucleotide substitutions in the first position of G-runs (codon changes HHG→HHH, HGG→HHG, GGG→HGG, where H is A, C or T). Two independent metrics were used for this purpose: the conservation index Cn, calculated on the basis of partition distances in a set of physicochemical properties of amino acid residues (Taylor, 1986; Livingstone, Barton, 1993), and the weights of amino acid substitutions in the BLOSUM62 matrix, compiled from several hundred groups of homologous proteins (Henikoff S., Henikoff J.G., 1992). Although G→A substitutions generally caused smaller changes in the properties and occurrence of amino acid residues, as expected for class-conserving point mutations, the difference from G→C and G→T substitutions was not statistically significant (Kruskal–Wallis test with Dunn’s correction for multiple comparisons, p > 0.05).

**Fig. 2.**
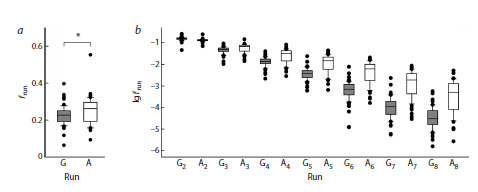
Length of polypurine runs in prokaryotic genomes. a, the total fraction of G or A in runs of any length in the respective purine nucleotide content in the genome (frun). * p <0.05 (Mann–Whitney test). b, the fraction of G or A in the runs 2 to 8 nucleotides long in the respective purine nucleotide content in the genome. In all cases, the difference between G-runs and A-runs is significant at p < 0.001 (Mann–Whitney test). Here and below, the line in the box marks the median, the boundaries of the box correspond to the first and third quartiles, the whiskers, to the 10th and 90th percentiles, and the dots are outliers.

All genomic sequences were searched for the HGnH and BAnB runs and the corresponding complementary-strand DCnD and VTnV runs (H = A, C or T; B = C, G or T; D = A, G or T; V = A, C or G) with the length n ≥ 2. The frequency of polypurine runs in the genomes was higher than that expected from a random nucleotide distribution with the same G+C composition (one-sample Wilcoxon test, p < 0.001), indicating the functional importance of such sequences. An increased frequency of substitutions at the first position of G-runs should gradually lead to their shortening. Indeed, when comparing the lengths of G-runs and A-runs in prokaryotic genomes, adjusted for the content of the respective purine nucleotides, it turned out that G-runs are, on average, shorter (Fig. 2a). In this case, HGG trinucleotides were more common than BAA, but in longer repeats, the frequency of A-runs was higher (Fig. 2b).

**Fig. 3.**
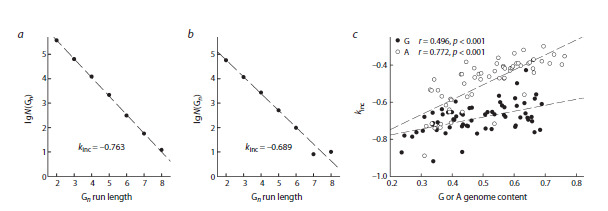
Dependence of the number of polypurine runs in prokaryotic genomes on the run length and the nucleotide composition of the genome. a, b, examples of the dependence of the number of G-runs N(Gn) on their length for the genomes of E. coli (a; genome size 4.64 × 106 bp, G+C content 51.0 %) and Ch. trachomatis (b; genome size 1.04 × 106 bp, G+C content 41.5 %). c, dependence of kinc on the nucleotide composition of the genome (G+C content for G-runs, A+T content for A-runs). Black dots, G-runs, white dots, A-runs; dashed lines show linear regressions with the regression coefficients indicated on the plot.

For a more detailed analysis of the run length distribution, we have studied the variability of their lengths in each genome. The number of G-runs and A-runs in each genome decreased almost strictly exponentially in the length range from 2 to 5–6. At n > 5–6, deviations in either direction were observed in some cases due to the small number of such runs, especially in small genomes (Fig. 3a, b). Using the linear portion of the relationship between the log of the number of repeats and run length, one can determine the increment coefficient kinc, which indicates how easily a run can be extended by one nucleotide in a genome with a given nucleotide composition: the higher the kinc, the greater the proportion of longer runs in the genome. When comparing the dependence of kinc for G-runs and Aruns in genomes of different composition, we have found that G-runs grow more slowly with increasing G+C content than A-runs grow with increasing A+T content (Fig. 3c). Thus, in prokaryotic genomes, the balance of G-run elongation and shortening, determined by many factors, is shifted towards shortening compared to A-runs.

**Fig. 4.**
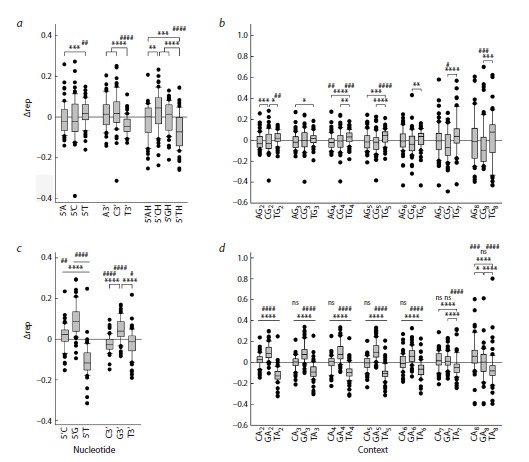
Representation of different 5’- and 3’-flanking nucleotides in polypurine runs. a, c, deviation from the frequency of 5’- and 3’-flanking nucleotides for G-runs (a) and A-runs (c) of any length expected on the basis of the content of the respective nucleotide in the genome. b, d, deviation from the frequency of 5’-flanking nucleotides in G-runs (b) and A-runs (d) 2–8 nucleotides long. Difference from expected: # p <0.05, ## p < 0.01, ### p < 0.005, #### p <0.001 (one-sample Wilcoxon test with Dunn’s correction for multiple comparisons); ns, no significant difference. Differences between groups: * p < 0.05, ** p < 0.01, *** p < 0.005, **** p < 0.001 (Kruskal–Wallis test with Dunn’s correction for multiple comparisons).

The lengths of polypurine runs can change in either direction due to DNA polymerase slippage during DNA synthesis (Kunkel, Bebenek, 2000) or selection based on the physicochemical properties of polypurine regions (Bansal et al., 2022), but these processes are independent of the nucleotides surrounding the run. In contrast, shortening of G-runs due to damage to the 5′-terminal base should be accompanied by a characteristic mutational spectrum determined by the properties of replicative DNA polymerases. Therefore, it was of interest to determine the extent to which the frequencies of 5′-flanking nucleotides differ from each other and from their overall abundance in the genome. To quantitatively characterize these differences, we have introduced the Δrep parameter representing the difference between the observed and expected frequency of each nucleotide. The frequency of T in the first position before G-runs was statistically significantly higher than expected and than the frequency of A and C (Fig. 4a). The frequency of A and C nucleotides in this position was slightly lower than expected, but this difference did not reach significance; their representation also did not differ from each other. T was more frequent than either A or C nucleotide at any G-run length, and its representation was higher than expected before G2, G4, G5, and G6 runs (Fig. 4b). A was underrepresented in this position only before G4 runs, and C was underrepresented before G5 and longer G-runs. In contrast, T was underrepresented both at the 3′-side of Gruns and at the second position from their 5′-side (Fig. 4a). Overall, these data support a model of preferential oxidation of the first G in the runs to 8-oxoG followed by G→T transversions.

Quite unexpectedly, the nucleotide distribution before A-runs was even more uneven than before G-runs. At this position, T was underrepresented, while C and G were overrepresented (Fig. 4c). For C, this deviation was explained primarily by overrepresentation of CAA trinucleotides, while for G, an increased frequency of occurrence was observed up to a run length of 6 nucleotides (Fig. 4d ). A decrease in the fraction of T also occurred in runs of any length (Fig. 4d ). After A-runs, the occurrence of C and T was lower than expected, while G was higher than expected (Fig. 4c). It is possible that these deviations can also be explained by DNA damage and subsequent DNA polymerases errors; however, the mechanistic reasons underlying such events remain unclear at present

**Fig. 5.**
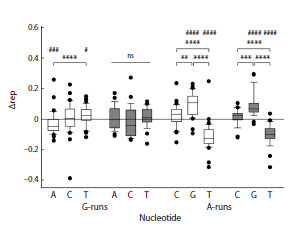
Representation of 5’-flanking nucleotides in polypurine runs in the genomes of aerobic (white) and anaerobic (gray) microorganisms.Deviation from the expected representation is shown for G-runs and A-runs of any length. Difference from expected: # p < 0.05, ### p < 0.005, #### p < 0.001 (one-sample Wilcoxon test with Dunn’s correction for multiple comparisons). Differences between groups: ** p < 0.01, *** p < 0.005, **** p < 0.001 (Kruskal– Wallis test with Dunn’s correction for multiple comparisons); ns, no significant difference

The amount of 8-oxoG generated in the genome directly depends on the presence of reactive oxygen species in the intracellular environment (Halliwell, Gutteridge, 2015). Prokaryotes are exceptionally diverse in their energy metabolism pathways: some follow a strictly anaerobic lifestyle, while others are obligate aerobes or facultative anaerobes and are subject to more intense oxidative stress. We have compared the statistics of the occurrence of 5′-flanking nucleotides of G-runs in the genomes of these two groups (Table 1). In aerobic prokaryotes, T was found at this position with an increased frequency compared to the expected, and A, with a decreased frequency (Fig. 5). For anaerobic microorganisms, no significant difference in the occurrence of 5′-flanking nucleotides was found (Fig. 5). However, when comparing the abundance of A, C and T directly between the aerobic and anaerobic groups, the differences did not reach statistical significance, which is most likely due to insufficient sample size. For A-runs, the difference in the occurrence of 5′-flanking nucleotides in the genomes of aerobes and anaerobes was the same as in the combined group (compare Fig. 4c and Fig. 5). Thus, the reduced level of oxidative stress in anaerobic microorganisms may be associated with a less pronounced predominance of T at the position flanking the 5′-side of G-runs; however, further research is required to answer this question more definitively.

Unlike those of prokaryotes, eukaryotic genomes are characterized by a large number of repetitive elements such as transposons, satellite and telomeric DNA, the precise sequences of which are inaccessible to traditional highthroughput sequencing methods (Richard et al., 2008; Liao et al., 2023). The advent of ultra-long sequencing (Oxford Nanopore, PacBio HiFi) has made it possible to fill these gaps. The recently published human genome read using a combination of methods with telomere-to-telomere (T2T) coverage and high quality (estimated telomeric error rate of ~ 4×10−8) (Nurk et al., 2022), provides the opportunity to analyze the context of G-runs without the distortions caused by a higher representation of unique sequences.

**Fig. 6.**
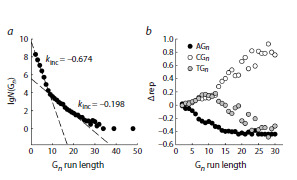
Dependence of the number of G-runs and the representation of the 5’-flanking nucleotides in the human genome on the run length. a, dependence of the number of G-runs N(Gn) on their length. The dashed lines correspond to the linear regression; the kinc values for n = 2–8 and n = 9–16 are shown on the plot. b, The dependence of Δrep of the 5’-flanking nucleotide (black, A, white, C, gray, T) on the run length.

The significantly larger size of the human genome compared to prokaryotic ones allowed us to identify interesting patterns in the distribution of Gn runs size. For n = 2–8, their number decreased exponentially and was described by an increment coefficient kinc = −0.674, which is very close to the center of the distribution of kinc values for G-runs in prokaryotes (compare Fig. 6a and Fig. 3c; z = 0.141). For n = 9–16, the exponential dependence was preserved, but the rate of decrease in the number of runs decelerated: the kinc value increased to −0.198, which lies far outside the range of kinc values for prokaryotic genomes (compare Fig. 6a and Fig. 3c; z = 5.97). Runs of this size were absent in prokaryotic genomes or were present in a handful of cases, so it was impossible to detect this transition. Further increase in the length of G-runs was accompanied by an even greater deceleration of the rate of decrease in their number (Fig. 6a). Obviously, around n = 8–9 (the breakpoint value determined by the piecewise regression method: n = 8.72 ± 0.04), the balance of G-run shortening and elongation is shifted in favor of the latter; run growth due to DNA polymerase slippage during replication or repair becomes self-sustaining, as in the well-studied case of trinucleotide repeat runs (Mirkin, 2007; McMurray, 2010).

An even more unexpected pattern emerged from the analysis of the frequency of 5′-flanking nucleotides. Since it is well known that the number of CG dinucleotides in the human genome is reduced due to their role in epigenetic regulation (Fazzari, Greally, 2004), the expected frequency was calculated based on the dinucleotide rather than the total nucleotide frequency. At n = 2, the nucleotide frequency closely matched the expected value, but then the Δrep values for A steadily decreased, while the representation of C and T, in contrast, increased at virtually the same rate (Fig. 6b). However, starting from n = 8–11 (the breakpoint value for Δrep(C)−Δrep(T), de0.6 termined by the piecewise regression method: n = 9.28 ± 1.10), the dependencies for C and T diverged sharply: the representation of T decreased, while the representation of C increased. One possible explanation for this phenomenon may be that longer G-runs serve as more effective traps for holes migrating along the DNA duplex leading to hyperoxidation of the 5′-terminal 8-oxoG to guanidinohydantoin and spiroiminodihydantoin with a corresponding switch in the preferential nucleotide substitutions from G→T to G→C.

Telomeric DNA is a distinct class of highly repetitive DNA in eukaryotic genomes, represented in humans by multiple copies of the TTAGGG hexanucleotide. Telomeric repeats are known to serve as hotspots for DNA oxidation to form 8-oxoG (Billard, Poncet, 2019; Opresko et al., 2025). Telomere ends in germline cells are elongated by telomerase, a specialized DNA polymerase that uses telomerase RNA as a template, so changes in these regions are not associated with damage to genomic DNA. However, even in the presence of active telomerase, the bulk of telomere length is replicated by the usual semiconservative mechanism (Pfeiffer, Lingner, 2013; Higa et al., 2017; Bonnell et al., 2021), which can lead to the accumulation of mutations in them. Thus, the telomere sequence in human somatic cells (in the case of the T2T genome, the immortalized telomerase-expressing CHM13hTERT chorionic cell line) reflects both their recent elongation by telomerase in germline cells and mutagenesis events in past generations and in individual development

**Fig. 7.**
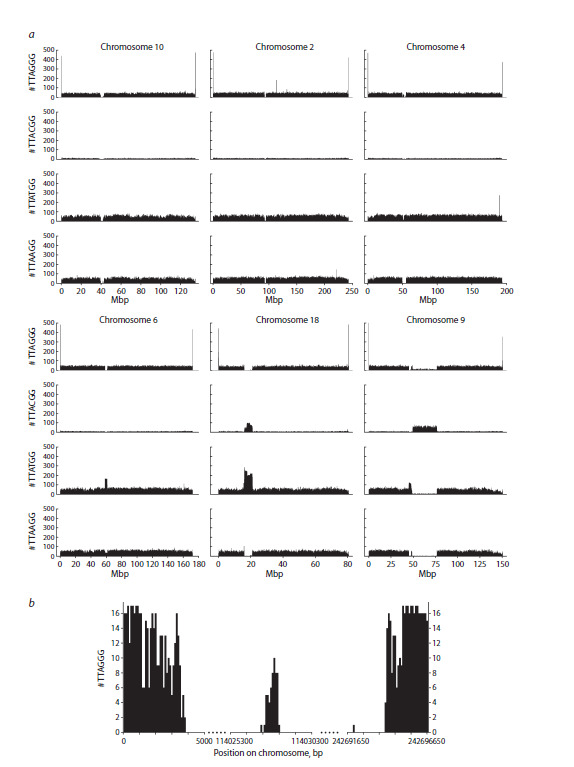
Examples of the distribution of TTAGGG, TTACGG, TTATGG, and TTAAGG repeats in human chromosomes. a, distribution of the repeats along the entire length of chromosomes 10, 2, 4, 6, 18, and 9. The number of repeats is calculated in 100-kb bins. b, distribution of TTAGGG repeats in telomeric regions and in the region of fusion of the ancestral telomeres on chromosome 2. The number of repeats is calculated in 100-bp bins.

The distribution of TTAGGG repeats in chromosomes (calculated for both DNA strands) had a fairly expected pattern, with frequency peaks at the ends of the chromosomes and a dip in the pericentromeric region (Fig. 7a). The only exception was chromosome 8, for which, on the contrary, a slight increase in the number of these repeats was observed in the centromere region. On chromosome 2, a peak in the frequency of telomeric repeats was clearly visible in the region of the fusion of two ancestral hominid chromosomes that formed the evolutionarily young human chromosome 2 (Ijdo et al., 1991; Fig. 7a). However, a more detailed analysis of this region shows that it has already significantly degraded, keeping far fewer TTAGGG repeats than in true telomeres (Fig. 7b). Interestingly, similar peaks were found on chromosomes 15 and 22 in the introns of the active protein-coding genes ATP10A and MICAL3; they may represent remnants of translocated telomere fragments.

TTAAGG, TTACGG, and TTATGG repeats were distributed across chromosomes without telomeric peaks. The overall frequency of TTACGG repeats was significantly lower than that of TTAAGG and TTATGG, consistent with the reduced abundance of CG dinucleotides in the human genome (Fig. 7a). Separate peaks in repeat frequency were observed on chromosome 2 for TTAAGG, chromosomes 8, 12, 17, and Y for TTACGG, and chromosomes 4 and 22 for TTATGG (Fig. 7a). A characteristic pattern of repeat distribution in the pericentromeric region with gaps in all TTANGG variants was observed for chromosomes 1–5, 7, 10–12, 16, 19, and 21. In other cases, one repeat type predominated in the centromere region, while others were depleted, with their combined deficiency compensating for the excess of the predominant type, as shown in Fig. 7a for chromosome 6. In chromosomes 6, 13–15, 22, and X, TTATGG was the predominant repeat; in chromosome 8, it was TTAGGG, and in chromosome 17, TTACGG. Chromosome 18 was distinguished by coinciding peaks in the distribution of two repeats, TTACGG and TTATGG (Fig. 7a). In the long arm of chromosome 9, in the region of constitutive heterochromatin adjacent to the pericentromeric region with an excess of TTATGG, there was a long stretch with a predominance of TTACGG.

Obviously, the cases of co-localization or oppositely phased localization of TTANGG repeats in non-telomeric regions are not due to point mutations in the TTAGGG repeat but reflect the presence of repeating elements containing one or two of these hexanucleotides in these loci. In contrast, point mutations in the first position of the G3-run of the telomeric repeat should be most obvious in the regions consisting mainly of TTAGGG, that is, in the telomeres proper and intrachromosomal blocks of telomere-like repeats. To analyze the frequency of substitutions in such regions, we have singled out the telomeric regions and intrachromosomal blocks where at least 100 copies of the TTAGGG repeat were found in 100-kb bins. They were divided into shorter 100-bp bins. A bin filled with only TTAGGG repeats corresponds to 16 or 17 copies (depending on the position of the first complete hexanucleotide in the bin). The bins containing at least 9 TTAGGG copies, accounting for more than half the bin length, were selected for analysis.

Counting the occurrence of TTAAGG, TTACGG, and TTATGG in the studied regions revealed clear significant enrichment of G→A substitutions at the first position of the G3-run compared to G→C and G→T substitutions (Table 2). In comparison with G→A, the total number of G→C and G→T changes was fivefold lower, and their frequencies did not differ significantly from each other. Thus, although telomeric repeats serve as preferential sites of guanine oxidation, this is not reflected in the increased frequency of G→T point mutations. The difference between the representation of A and C+T at the 5′-flanking position of GG dinucleotides between telomeric repeats and the rest of the genome may indicate the existence of a mutational process in telomeres that is distinct from G oxidation at the 5′-position of GGG.

**Table 2.**
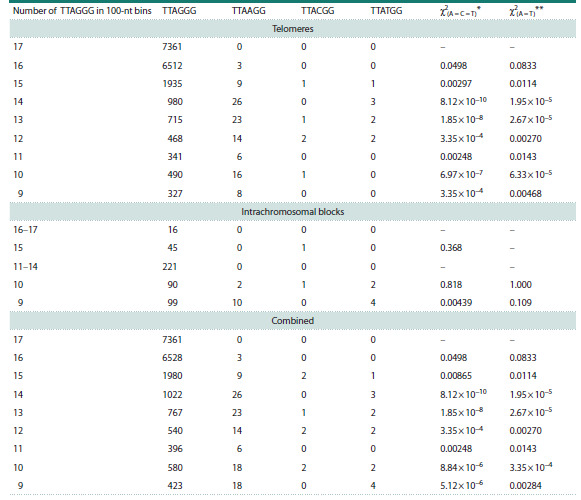
Representation of TTANGG in telomeres and intrachromosomal blocks of telomere-like repeats * χ2 values for the null hypothesis of equal representation of A, C and T. ** χ2 values for the null hypothesis of equal representation of A and T.

## Conclusion

In conclusion, the analysis of the nucleotide context of Gruns in a set of 62 complete prokaryotic genomes and in the human T2T genome revealed that the representation of T at the position adjacent to G-runs is generally increased, which is consistent with the model of G oxidation at the 5′-position of the runs followed by G→T mutations. Other patterns in the distribution of 5′-flanking nucleotides were also identified: uneven nucleotide frequency at the position adjacent to A-runs, increased representation of C at the 5′-side of long G-runs in the human genome, and the predominance of G→A substitutions at the 5′-position in telomeric repeats. The hypothesis that G-run elongation may lead to a shift in the specificity of single-nucleotide mutations from G→T to G→C due to a change in the nature of the precursor lesion can be tested experimentally. The characteristic mutation spectrum in telomeric repeats may be caused by their tendency to fold into G-quadruplex structures, which hinder the movement of DNA polymerases (Pfeiffer, Lingner, 2013; Higa et al., 2017; Bonnell et al., 2021), but this proposal requires a detailed study of the fidelity of human replicative DNA polymerases on intact and damaged templates of this structure. For A-runs, the existence of preferential sites of DNA damage is not known; given that A-runs are longer than G-runs (Fig. 2), the difference in the relative representation of C, G, and T in the 5′-flanking position may not be associated with the mutational process. The explanation of all these identified patterns requires further research.

## Conflict of interest

The authors declare no conflict of interest.
